# A Rare Case of Isolated Type III Salter-Harris Physeal Injury of the Distal Ulna

**DOI:** 10.7759/cureus.14547

**Published:** 2021-04-18

**Authors:** Vivek Tiwari, Samir Dwidmuthe, Samrat S Sahoo

**Affiliations:** 1 Department of Orthopedics, All India Institute of Medical Sciences, Nagpur, IND

**Keywords:** distal ulna, salter-harris injury, ulnar physis, physeal arrest, physeal injury

## Abstract

Pediatric distal forearm fractures are very commonly seen in orthopedic and trauma services. The majority of such fractures involve either distal radius alone or concomitant with distal ulna physis. Isolated injuries of the distal ulna physis are exceedingly rare. Moreover, only one case of isolated Salter-Harris type III distal ulnar physeal injury has been reported in English literature. We report the second such case of isolated distal ulnar physeal injury with Salter-Harris type III pattern in a 15-year-old male child. The injury was treated conservatively in a short-arm splint for four weeks with a good outcome. At the last follow-up of one year, there was no physeal abnormality or growth disturbance detected. Such injuries need to be ruled out in all pediatric distal forearm fractures and can be managed conservatively, if undisplaced, with good outcomes. These patients also need regular monitoring till the closure of the physis to look for the development of any ulnar shortening or deformity due to physeal growth arrest.

## Introduction

Injuries of the distal forearm are quite common in childhood and adolescence [1]. Most of these injuries occur as a result of a fall on the outstretched hand [2]. Such injuries usually result in either non-physeal injuries including Torus fractures, distal radius physeal injury alone, or distal radius combined with distal ulna physeal injury. Isolated distal ulna physeal injury is quite rare. We report a case of distal ulna type III Salter-Harris physeal injury in an adolescent patient, which was treated conservatively with good outcomes at the last follow-up of one year [3].

## Case presentation

**Figure 1:**
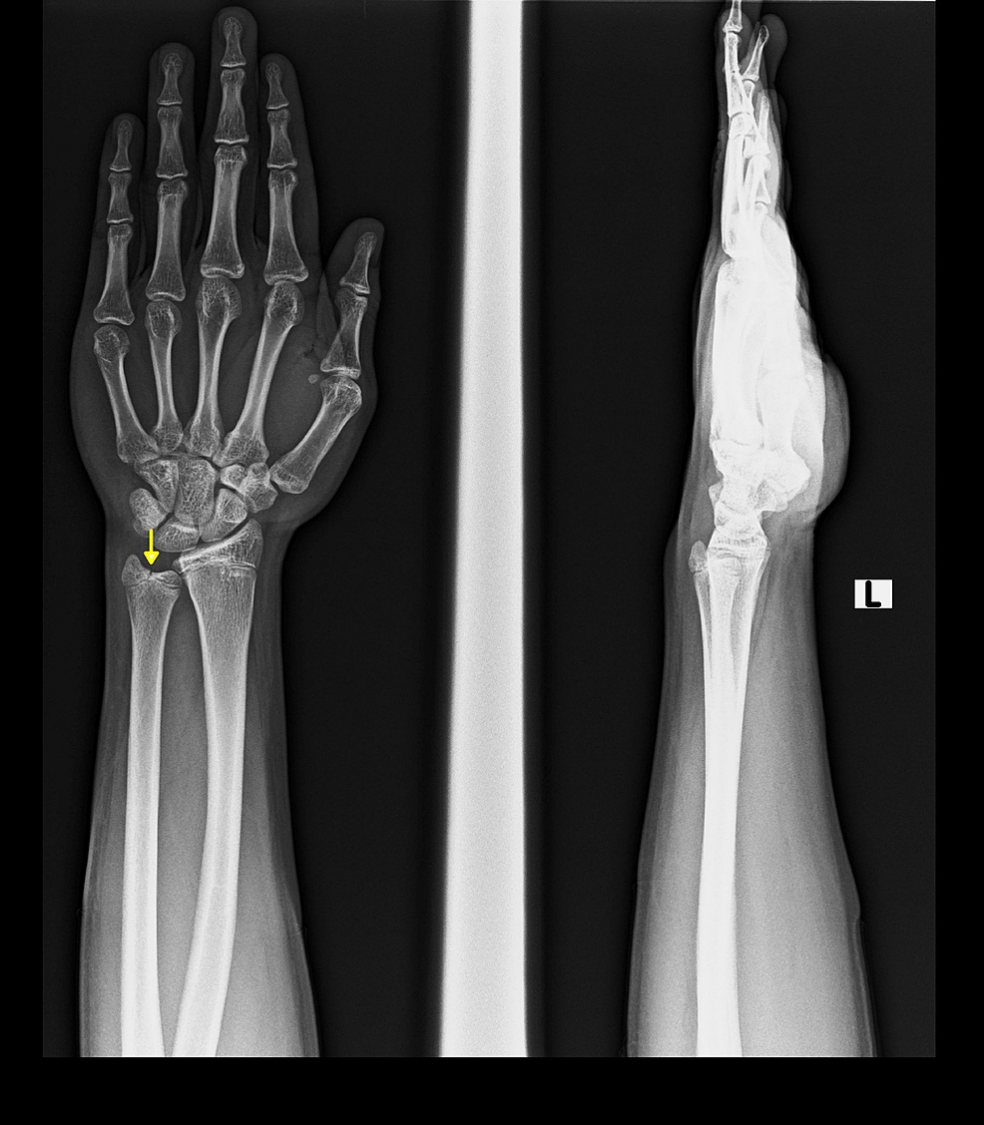
Plain radiograph of left wrist with forearm - anteroposterior and lateral views A linear vertical undisplaced fracture line in the distal ulnar epiphysis (yellow arrow).

A 15-year-old boy presented to the orthopedic outpatient department of our institute after falling from six feet height on his left hand (non-dominant hand) while playing. He complained of pain in the ulnar aspect of the left wrist and difficulty in holding objects. There was no history of injury to any other part of the body. On examination, he was found to have tenderness and swelling over the left distal ulna, and the wrist range of movement was painful. There was no tenderness over the distal radius or the distal radio-ulnar joint. There were no signs of distal radio-ulnar joint instability. There were no clinical signs suggestive of triangular fibrocartilage complex (TFCC) injury. Plain radiograph of the left wrist with forearm showed negative ulnar variance and a linear vertical undisplaced fracture line in the epiphysis of distal ulna without any physeal abnormality, categorizing it as a type III Salter-Harris injury of distal ulnar physis (Figure 1).

**Figure 2:**
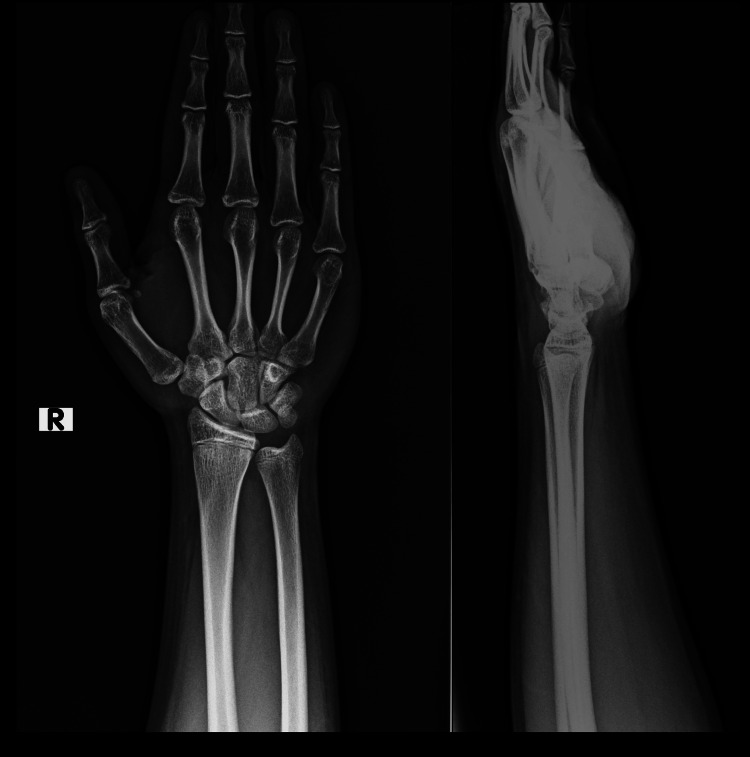
Plain radiograph of right wrist with forearm - anteroposterior and lateral views

X-ray of the contralateral limb was also done for comparison; it was found unremarkable except for negative ulnar variance (Figure 2).

**Figure 3:**
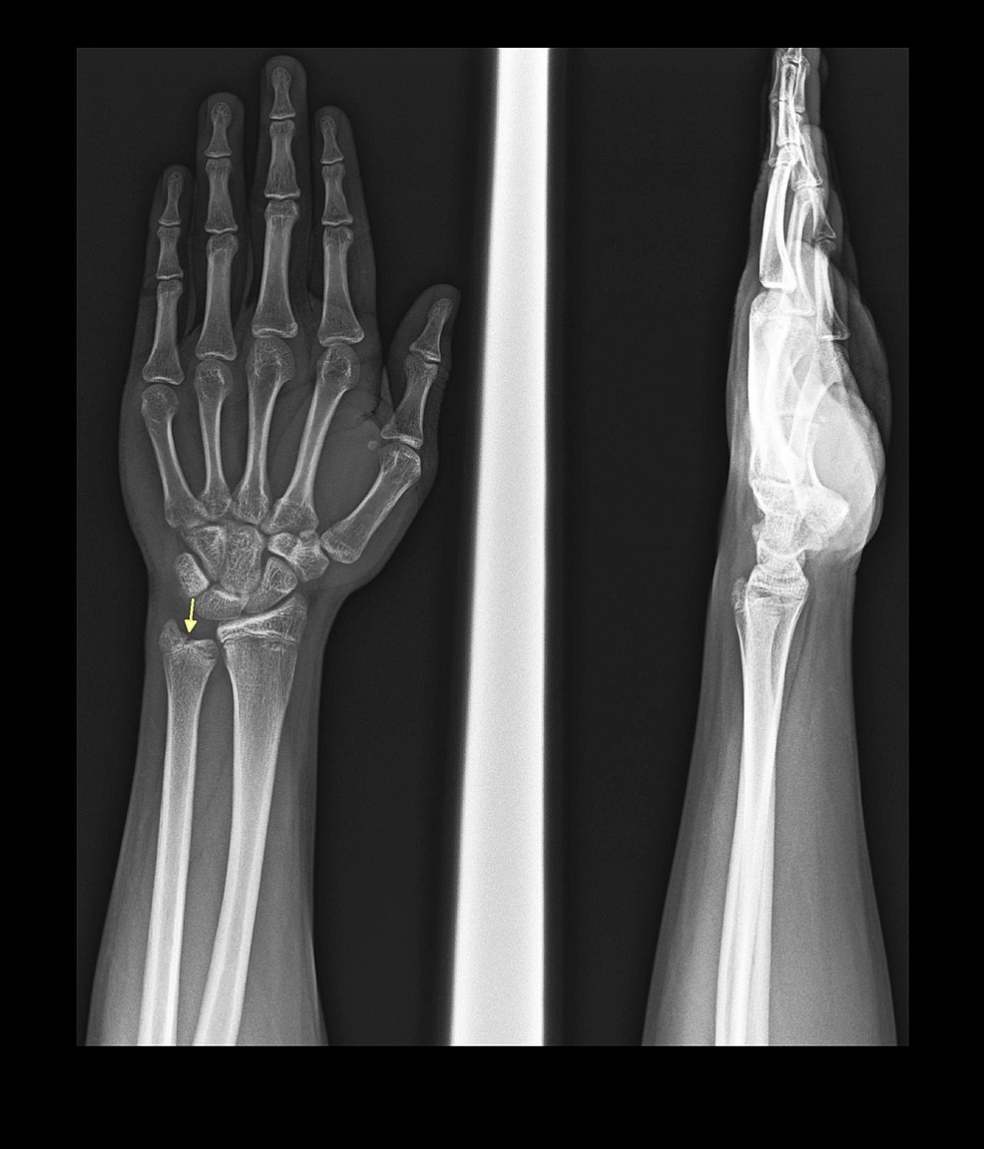
One-month follow-up plain radiograph Partial healing of the fracture (yellow arrow).

The fracture was managed in a below-elbow splint for four weeks. After four weeks, there was no tenderness over the distal ulna. The follow-up plain radiograph at four weeks demonstrated partial healing of the fracture (Figure 3).

**Figure 4:**
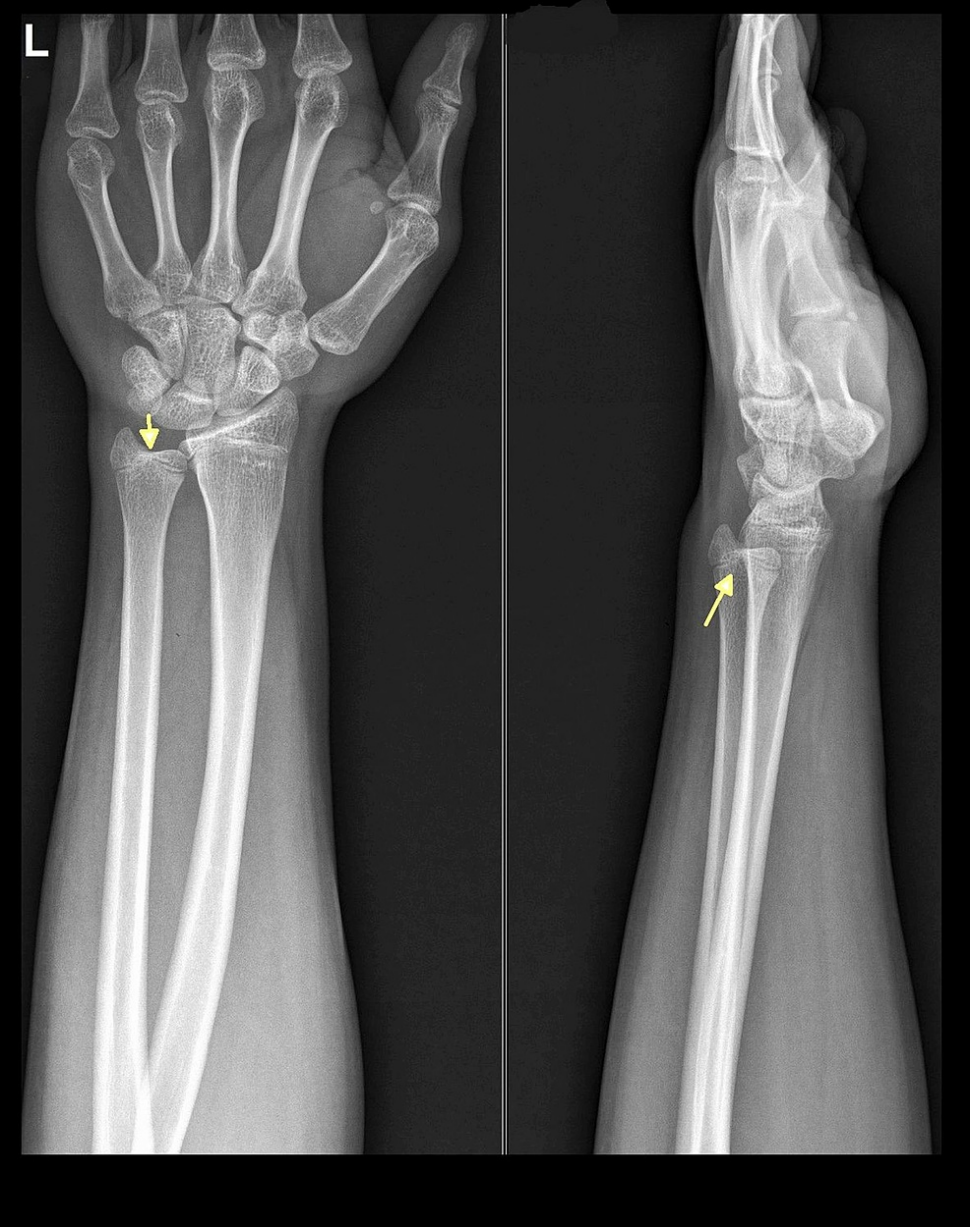
One-year follow-up plain radiograph Complete healing of the fracture without any growth arrest/deformity (yellow arrows).

He resumed wrist range of motion exercises and gentle work using the left hand after four weeks. At the last follow-up of one year, he was pain-free without any symptoms with Disabilities of the Arm, Shoulder, and Hand (DASH) score of 7.5 and a Patient-Rated Wrist Evaluation (PRWE) score of 6 [4,5]. The one-year follow-up plain radiograph demonstrated complete healing of the fracture without any growth arrest/deformity (Figure 4).

## Discussion

Pediatric orthopedic injuries commonly involve the distal forearm [6]. The majority of such fractures include only distal radius or concomitant with the distal ulna. Isolated injuries of the distal ulna physis are quite rare. Evans et al. reported a case of isolated injury involving distal ulnar physis, a Salter-Harris type II injury, which was irreducible due to interpolation of extensor carpi ulnaris tendon [7]. The fracture required open reduction through a direct ulnar approach and pinning. Golz et al. reported a case series of 18 patients and two traumatic amputation specimens with distal ulnar physeal injuries [8]. All such ulnar fractures were associated with concomitant distal radius fractures; the majority of the ulnar injuries, 10 in total, were Salter-Harris type I, and six of them were type III. In their series, four patients underwent operative reduction, and 55% had premature physeal closure. Cannata et al. reviewed long-term prognosis in 163 distal forearm physeal lesions including six distal ulnar physeal injuries [9]. Among them, only one patient had isolated distal ulnar physeal lesion, and none of the ulnar physeal injuries were Salter-Harris type III. They reported limb shortening of more than 1 cm in 50% of the distal ulnar physeal injuries after conservative management at a mean follow-up of 25.5 years; however, none of the patients had a severe functional disability.

Physeal injuries involving the distal radius are often associated with distal radio-ulnar joint injury as well. In patients where the physes are still open, such disruption of the distal radio-ulnar joint is often seen as distal ulnar physeal separation with associated epiphysiolysis of the distal ulna. Such lesions have been described as Galeazzi-equivalent fractures [10].

Isolated distal ulnar physeal injuries are extremely rare to find in English literature [8,9]. Suthar and Kothari described a rare case of distal ulnar physeal separation (Salter-Harris type I injury) - a Galeazzi-equivalent lesion along with splitting of epiphysis into two fragments (Salter-Harris type III injury) - along with distal radial metaphyseal injury, in a 13-year-old male patient [11]. The said case was treated with closed reduction and pinning for distal radial injury and open reduction and K-wire fixation for the ulna fracture, with a good outcome.

There has been only one case of isolated type III Salter-Harris injury of distal ulnar physis described in English literature. Yukata et al. described a rare case of isolated type III Salter-Harris injury of distal ulna in a 13-year-old patient, treated conservatively with good outcome and without any physeal arrest or other complications [12]. Our patient also had a similar pattern of injury without any concomitant distal radial injury. The patient was treated conservatively in a short-arm splint with good outcomes. At the last follow-up of one year, there was no physeal abnormality or growth disturbance detected; we plan to follow up with the patient till skeletal maturity to look for any ulnar shortening. Such injuries need to be ruled out in all pediatric distal forearm fractures and, when undisplaced, can be managed conservatively with good outcomes. These patients also need monitoring till the closure of the physis to look for any ulnar shortening or deformity due to physeal growth arrest.

## Conclusions

Distal forearm injuries, being one of the common trauma cases in the pediatric age group, need to be diagnosed and managed promptly and effectively. We reported a very rare variety of such injuries - an isolated type III Salter-Harris physeal injury involving distal ulna. This is the only second such case reported in English literature. Identification and appropriate management of these fractures hold paramount significance as their mismanagement can lead to physeal arrest/deformity. Such injuries should not be missed upon presentation and can have good outcomes with conservative management. Also, these patients need to be followed up till skeletal maturity to identify any evolving growth-related complications.
